# 原发性肺癌发生气管支气管转移的调查分析

**DOI:** 10.3779/j.issn.1009-3419.2020.101.15

**Published:** 2020-03-20

**Authors:** 明 路, 翔 朱, 宝山 曹, 宁 沈

**Affiliations:** 1 100191 北京，北京大学第三医院呼吸与危重症医学科 Department of Respiratory and Critical Care Medicine, Peking University Third Hospital, Beijing 100191, China; 2 100191 北京，北京大学第三医院病理科 Department of Pathology, Peking University Third Hospital, Beijing 100191, China; 3 100191 北京，北京大学基础医学院病理学系 Department of Pathology, Peking University, Health Science Center, Beijing 100191, China; 4 100191 北京，北京大学第三医院肿瘤化疗与放射病科 Department of Medical Oncology and Radiation Sickness, Peking University Third Hospital, Beijing 100191, China

**Keywords:** 肺肿瘤, 肿瘤转移, 气管肿瘤, 支气管肿瘤, Lung neoplasms, Neoplasm metastasis, Tracheal neoplasms, Bronchial neoplasms

## Abstract

**背景与目的:**

气管支气管转移（endotracheal and endobronchial metastases, EEM）在肺癌中罕见，国外文献报道可发生于手术切除后，但国内目前尚未见相关报道，本研究旨在总结和分析肺癌发生EEM的临床特征。

**方法:**

回顾2015年1月-2018年12月于北京大学第三医院确诊原发性肺癌并行支气管镜的患者，同时检索截至2020年2月PubMed检索系统中的病例，采集并比较两组患者的临床、病理、影像、支气管镜和预后等资料。

**结果:**

我院共有6例肺癌伴EEM入选，发生率为0.62%（6/967），均为初诊为肺癌时即伴有EEM。鳞癌4例，腺癌1例，小细胞肺癌1例。Ⅲb期1例，Ⅳ期5例。中央型肺癌5例，周围型1例。EEM在支气管镜下表现为肺癌原发灶之外的气道黏膜结节或息肉性病变5例、局灶性黏膜异常1例。转移至对侧支气管5例，至同侧支气管和气管各1例。中位总生存期为7.5个月。从PubMed数据库共检索到13例，其中12例为肺癌术后随诊胸部计算机断层扫描（computed tomography, CT）异常继而确诊为EEM。中央型9例，鳞癌8例，EEM在CT上表现为腔内结节10例，气管壁局限增厚2例，支气管镜下均表现为气道黏膜结节或息肉样病变。转移至气管10例，至对侧支气管5例，至同侧支气管1例。

**结论:**

EEM是原发性肺癌罕见的转移方式，可发生于初诊时，也可发生于术后，多见于晚期中央型鳞癌，预后差。

## Abstract (en)

**Background and objective:**

Endotracheal and endobronchial metastases (EEM) is a rare manifestation in primary lung cancer. It has not yet been reported in Chinese literatures. The aim of this study was to summarize and analyze the clinical feature of lung cancer with EEM.

**Methods:**

We retrospectively reviewed 6 patients who presented with EEM of lung cancer from Peking University Third Hospital from January 2015 to December 2018. With "endotracheal metastases, endobronchial metastases, lung cancer" as the keywords, 13 cases were retrieved from PubMed database until February 2020. The clinical, radiologic and bronchoscopic data were collected.

**Results:**

Six patients were selected from 967 patients with lung cancer, and all were diagnosed with lung cancer and EEM simultaneously. There were 4 cases of squamous cell carcinoma, 1 case of adenocarcinoma, and 1 case of small cell lung cancer. One patient had stage Ⅲb and 5 patients had stage Ⅳ. There were 5 cases of central lung cancer and 1 case of peripheral lung cancer. EEM on bronchoscope examination presented as endoluminal nodular or polypoid lesion in 5 patients, and abnormal white bulge in 1 patient. 5 cases metastasized to the contralateral bronchus, 1 case to the ipsilateral bronchus and 1 case to the trachea. The median overall survival was 7.5 months. Totally 13 cases of lung cancer with EEM were retrieved from PubMed database. 12 cases were diagnosed during the follow up after lung cancer resection. There were 8 cases of squamous cell carcinoma and 9 cases of central type. Endotracheal or endobronchial nodules showed in 10 cases and eccentric wall thickening in 2 cases were seen on chest computed tomography (CT), which corresponding to the nodular or polypoid lesion bronchoscopically. 5 cases metastasized to the contralateral bronchus, 10 cases to the trachea and 1 case to the ipsilateral bronchus.

**Conclusions:**

EEM is a rare metastasis of lung cancer, which can occur at the initial diagnosis of lung cancer or after surgical resection. It is often seen in the patients of squamous cell carcinoma with central type in advanced stage. The prognosis is poor.

恶性肿瘤转移包括直接蔓延、淋巴转移、血行转移和种植转移等四种经典方式。根据各器官自身的特性，近年来有学者提出了新的肿瘤转移方式。支气管和肺不仅富含血流和淋巴组织，还是气体传导器官，肺癌细胞可以微乳头结构、小实性癌巢或单个细胞的形式出现在远离主要肿瘤病灶的肺组织内，即肿瘤沿气腔播散（spread through air spaces, STAS）^[1-3]^，2015年正式被世界卫生组织（World Health Organization, WHO）肺肿瘤组织认可，这意味着我们已经接受了肿瘤可通过“气体”侵袭转移的新概念^[4]^。

很多肺外实体瘤，包括肾癌、结肠癌、乳腺癌、食管癌、舌癌、骨巨细胞瘤、宫颈癌、皮肤基底细胞癌等可转移至气管和/或支气管管壁^[5-8]^，有学者称之为支气管转移（endobronchial metastases, EBM）^[6]^或气管和支气管转移（endotracheal and endobronchial metastases, EEM）^[7, 8]^。

关于原发性肺癌发生EEM，国外文献显示EEM可发生于肺癌手术切除后，而目前国内尚未见相关报道。我们曾发现1例最初确诊小细胞肺癌同时即发生跳跃性气管和支气管转移的病例^[9]^，现进一步回顾性分析2015年1月-2018年12月在北京大学第三医院呼吸科确诊的6例原发性肺癌伴EEM的患者，同时检索国外文献报道的病例，总结此类患者的临床、病理、影像、支气管镜表现和预后。

## 1 对象与方法

### 1.1 研究对象

回顾2015年1月-2018年12月北京大学第三医院呼吸与危重症医学科确诊为肺癌的所有住院病例。纳入标准：①组织病理证实为原发性肺癌；②于我院行支气管镜检查，符合肺癌EEM的诊断标准，即：在肺癌主体病灶之外的气管和/或支气管黏膜出现转移癌，支气管镜下可见段级以上支气管或气管存在结节、息肉样等黏膜异常病变，但又和原发性肺癌主体病灶无解剖连续性，且与肺癌主体病灶病理类型相同。由原发肿瘤直接侵犯邻近支气管或气管，主体病灶和转移灶相连续者，不属于该范畴；③临床、影像和病理学资料和随访完整。筛查患者967例，共6例符合上述标准。同时，通过PubMed检索系统以“lung cancer、endotracheal metastases、endobronchial metastases”为检索词，时限为1967年1月-2020年2月，共检索到符合要求的9篇英文文献中的13个病例^[10-18]^。

### 1.2 资料收集

收集所有患者的临床资料，包括年龄、性别、肿瘤原发灶-淋巴结-转移（tumor-node-metastasis, TNM）分期、胸部影像和支气管镜结果、病理类型、基因[包括表皮生长因子受体（epidermal growth factor receptor, *EGFR*）/间变性淋巴瘤激酶（anaplastic lymphoma kinase, *ALK*）/ROS原癌基因1受体酪氨酸激酶（ROS proto-oncogene 1 receptor tyrosine kinase, *ROS1*）]突变以及总生存期。

### 1.3 我院和文献报道的肺癌发生EEM患者的临床特征比较

比较两组患者的性别、年龄、原发性肺癌的位置、病理类型以及EEM的数量和位置等。

### 1.4 统计学方法

应用SPSS 19.0统计学软件进行分析。统计差异运用*Fisher*精确检验，当*P* < 0.05为差异有统计学意义。

## 2 结果

### 2.1 我院的肺癌发生EEM患者的临床特征

共6例原发性肺癌发生EEM的患者入组本研究（表 1），总发生率为0.62%（6/967），均为最初确诊肺癌同时发现EEM。中位年龄64.5岁（57岁-85岁），男性4例。中央型肺癌5例，周围型1例[病例3，肺癌原发病灶由计算机断层扫描（computed tomography, CT）引导下肺穿刺活检证实]。鳞癌4例，腺癌1例，小细胞肺癌1例。基因检测（*EGFR*/*ALK*/*ROS1*）未见突变。肺癌TNM分期，Ⅲb期1例，Ⅳ期5例，所有患者区域淋巴结转移分期均为N3。EEM病灶在胸部CT上表现为气管支气管腔内结节3例和支气管管腔狭窄1例，有2例EEM在CT上不可见。支气管镜下EEM表现为局灶性黏膜异常1例（图 1），表现为气道黏膜结节或息肉性病变5例（图 2）。单发转移5例（图 1），多发转移1例（图 2），转移至气管1例（图 2），转移至肺癌原发灶的对侧支气管5例[包括主支气管3例，下叶支气管2例（图 1），上叶支气管1例]，转移至同侧支气管和气管各1例。中位总生存期为7.5个月（5个月-13个月）。

**1:** 我院6例原发性肺癌伴气管支气管转移的临床特征 Clinical features of six patients with EEM of primary lung cancer patients in our hospital

Case	Age/gender	Primary lung cancer			EEM bronchoscopic findings	OS (mo)
		Location	Pathology	Stage		
1	69/F	RLL	Squamous	Ⅳ (cT2N3M1)	Abnormal white bulge in the apical segment of LLL	8
2	64/M	RUL	Squamous	Ⅲb (cT2N3M0)	Small nodule in the apical segment of LLL	13
3	57/M	RUL	Squamous	Ⅳ (cT1cN3M1)	Multiple nodules in the left LM	7
4	65/M	LUL	Squamous	Ⅳ (cT2N3M1)	Polypoid nodule in the RUL	7
5	58/F	LUL	Adeno	Ⅳ (cT2N3M1)	Multiple nodules in the RM	10
6	85/M	LUL	SCLC	Ⅳ (cT2N3M1)	Small nodules in the trachea and LM	5
Adeno: adenocarcinoma; Squamous: squamous cell carcinoma; LLL: left lower lobe; LM: left main bronchus; LUL: left upper lobe; OS: overall survival; RLL: right lower lobe; RM: right main bronchus; RUL: right upper lobe; SCLC: small cell lung cancer; EEM: endotracheal and endobronchial metastases; F: female; M: male.						

**1:**
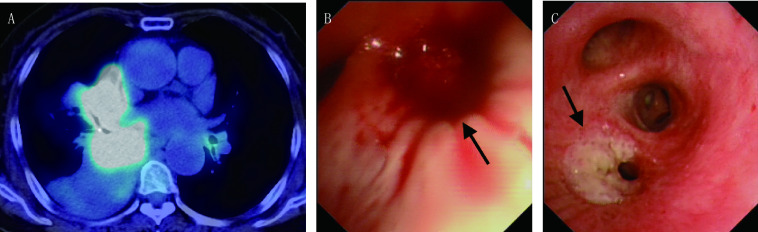
鳞癌患者发生EEM的胸部影像和支气管镜表现（表 1中患者1）。A：PET/CT示右肺门不规则软组织密度肿物，FDG摄取明显增高，伴右中下叶肺不张；左肺下叶背段支气管开口处FDG摄取轻度增高；B：支气管镜示右肺下叶支气管新生物伴出血（箭头）；C：左肺下叶背段支气管开口处可见局限性异常白色黏膜隆起（箭头） Chest radiographic and bronchoscopic images in a patient with squamous lung cancer and EEM (patient 1 in Tab 1). A: PET/CT showing irregular mass in the right hilum, with marked FDG uptake; B: Bronchoscopic examination showing an endobronchial tumor with bleeding in the right lower lobe (arrow); C: A white bulge with distinct border at the orifice of apical segmental bronchus in left lower lobe (arrow). FDG: F-18 fluorodeoxyglucose; PET/CT: positron emission tomography/computed tomography

**2:**
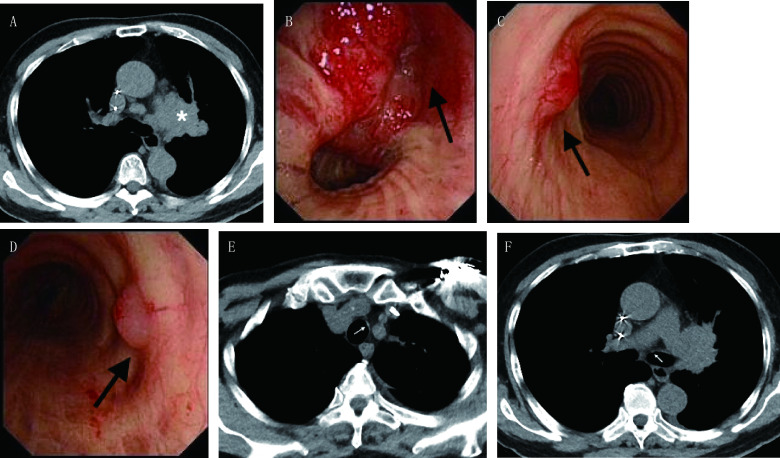
小细胞肺癌患者发生EEM的胸部影像和支气管镜表现（表 1中患者6）。A：CT示左肺门肿块（星号）；B、C、D：支气管镜示左肺上叶可见新生物致管腔完全闭塞（B箭头），气管上段左外侧壁息肉样病变（C箭头），左主支气管入口左侧壁息肉样病变（D箭头）；E、F：回顾肺CT分别可见气管和左主支气管局部管壁增厚（箭头） Chest radiographic and bronchoscopic images in a patient with SCLC and EEM (patient 6 in Tab 1). A: Chest CT showing an irregular mass in the left perihilar lung; B, C, D: Bronchoscopy revealing the left upper lobe bronchus was occluded completely by an endobronchial neoplasm (B, arrow), one polypoid lesion located in the left lateral wall of the upper trachea (C, arrow), and the other polypoid lesion in the medial wall of the left main bronchus inlet (D, arrow); E, F: Review of his chest CT showing eccentric thickening of the upper trachea (E, arrow) and the left main bronchus (F, arrow)

### 2.2 文献报道的肺癌发生EEM患者的临床特征

13例患者中，仅1例是在最初确诊小细胞肺癌同时发现的EEM，其余12例则是在肺癌切除术后随访8个月-52个月中发生的EEM。鳞癌8例，腺癌3例，小细胞肺癌2例。仅有1例腺癌患者接受了基因检测，为20外显子插入突变。在肺癌术后发生EEM的12例患者中（表 2），平均年龄58.5岁（44岁-78岁），男性8例。术后病理分期：Ia期2例，Ib期5例，IIa期1例，IIb期2例，Ⅲa期2例，发生EEM后未再进行全身评估和TNM分期。其中3例因呼吸道症状（2例咯血，1例咳嗽喘息）就诊而被发现，另9例均无症状，因术后常规CT检查而发现气管支气管异常。12例患者中，中央型肺癌9例，周围型3例。EEM病灶在胸部CT上表现为气管支气管腔内结节10例和管壁增厚2例，支气管镜下EEM均表现为气道黏膜大小不等的结节或息肉样病变。单发转移8例，多发转移4例。转移至气管10例，转移至肺癌原发灶的对侧支气管5例（均为左主支气管），转移至同侧支气管1例（右中叶）。

**2:** 文献报道的13例原发性肺癌伴气管支气管转移的临床特征 Clinical features of 13 patients with EEM of primary lung cancer patients reported in the literature

Case	Age/ gender	Primary lung cancer			Recurrence interval (mo)	EEM computed tomography or bronchoscopic findings
		Location	Pathology	Stage		
1^[10]^	63/M	RLL	Adeno	Ib	20	Polypoid lesions in RML and LM
2^[11]^	60/M	LUL	Adeno	Ib	26	Nodule in trachea
3^[11]^	68/M	LUL	Squamous	IIb	17	Nodule in trachea
4^[11]^	53/M	RLL	Squamous	Ia	52	Nodule in trachea
5^[11]^	66/M	RLL	Squamous	Ⅲa	8	Nodule in trachea
6^[11]^	53/M	RLL	Squamous	Ⅲa	11	Nodule in trachea
7^[11]^	64/M	LLL	Squamous	Ib	41	Nodule in trachea
8^[12]^	44/F	RML, RLL	Squamous	Ib	10	Multiple nodules in trachea and LM
9^[13]^	51/M	RML	Squamous	IIb	12	Polypoid lesions in trachea and LM
10^[14]^	57/F	RUL	Adeno	Ia	7	Polypoid lesions in trachea and LM
11^[15]^	46/M	RUL	Squamous	IIa	36	Multiple nodules in LM
12^[16]^	78/F	RUL	SCLC	Ib	12	Multiple nodules in trachea and bilateral main bronchi
13^[17]^	45/F	LLL	SCLC	NA	0	Polypoid lesions in trachea
NA: not available.							

### 2.3 两组患者临床特征比较

两组患者的性别、年龄、病理类型、肿瘤原发灶位置、转移灶数量均无统计学差异（*P* > 0.05），均为鳞癌多见，中央型肺癌多见，单发转移灶多见。我院确诊肺癌同时发现EEM的患者中，转移至左右主支气管较多，但两组差异无统计学意义。而文献报道的肺癌切除术后发生EEM的患者中，气管是EEM的最常见部位（*P* < 0.05）（表 3）。

**3:** 我院的6例原发性肺癌伴EEM和文献报道的12例肺癌术后发生EEM患者的临床特征比较 Comparison of 6 patients of primary lung cancer with EEM in our hospital with 12 cases of surgically resected lung cancer with EEM reported in the literature

Variables	Primary lung cancer with EEM (*n*=6)	Patients of surgically resected lung cancer with EEM (*n*=12)	*P*
Age (yr)			0.620
< 65	3 (50.0%)	9 (75.0%)	
≥65	3 (50.0%)	3 (25.0%)	
Gender			> 0.999
Male	4 (66.7%)	9 (75.0%)	
Female	2 (33.3%)	3 (25.0%)	
Pathology			
Squamous	4 (66.7%)	8 (66.7%)	> 0.999
Adeno	1 (16.7%)	3 (25.0%)	> 0.999
SCLC	1 (16.7%)	1 (8.3%)	> 0.999
Location of the primary lung cancer			> 0.999
Central type	5 (83.3%)	9 (75.0%)	
Peripheral type	1 (16.7%)	3 (25.0%)	
EEM			0.600
Simple	5 (83.3%)	7 (58.3%)	
Multiple	1 (16.7%)	5 (41.6%)	
Location of EEM			
Trachea	1 (16.7%)	10 (83.3%)	0.013
Main bronchus	3 (50.0%)	6 (50.0%)	> 0.999
Upper lobe	1 (16.7%)	0 (0.0%)	0.333
Middle lobe	0 (0.0%)	1 (8.3%)	> 0.999
Lower lobe	2 (33.3%)	0 (0.0%)	0.098

## 3 讨论

Brailey等^[19]^最早于1948年报道过肾癌发生支气管转移。此后，关于恶性肿瘤发生EEM的文献陆续报道，但绝大多数见于肺外肿瘤^[5-8]^，其发生率约为2%-28%^[6, 20]^，相比而言，原发性肺癌发生EEM的文献则很少，绝大多数为个案报道。不同研究方法得出肺癌EEM的发生率差异很大。Chong等^[11]^的报道显示，1, 372例非小细胞肺癌患者术后常规随访，共6例（0.44%）患者发生EEM，明显低于肺外肿瘤EEM的发生率。而苏格兰的一项尸检结果^[19]^显示，96例未行手术切除的肺癌患者，有17例（17.7%）可见EEM。目前国内尚未见肺癌EEM的相关报道。总结我们的6例患者发现，在最初确诊肺癌时即发生EEM者，临床罕见，总发生率为0.62%（6/967），这与Chong等^[11]^的报道结果相似。

EEM是肺癌的一种独立的特殊转移形式，还是晚期肺癌必然发生的终末期转移表现，目前尚不清楚。我们的6例患者，包括Ⅲb期1例和Ⅳ期5例，中位总生存期为7.5个月，这提示发生EEM多见于肺癌晚期，预后很差^[21]^。但苏格兰的尸检结果^[19]^显示，未行手术切除的肺癌患者尸检时仅17.7%存在EEM，这说明并非所有肺癌患者在终末期都会发生EEM。

关于EEM的具体机制，目前尚不清楚，有以下几种可能：①Chong等^[11]^报道了6例肺癌患者术后发生的气管转移，病理显示肿瘤均累及黏膜下层。其中1例在黏膜下淋巴管内见到癌栓，这为癌细胞通过黏膜下淋巴管发生EEM提供了一定的依据。本文的6例患者TNM分期淋巴结转移均为N3，达对侧肺门淋巴结和/或锁骨上淋巴结，与之相符。②肿瘤沿气腔播散（spread through air space, STAS）是指肺癌肿块的癌细胞可通过肺泡和毛细支气管通道播散到肺癌周边部分肺实质内，STAS的存在可导致术后总生存率降低，术后复发率增高^[1, 2]^。按照肿瘤可通过“气体”侵袭转移的这一思路，如果癌细胞是一个一个地向外播散，则只能在显微镜下才可见，在CT和支气管镜下不可见。但如果癌细胞随着呼吸或咳嗽以多个或较大的实性癌巢形式播散，发生EEM也并不是没有可能。

无论是我们的6例患者，还是文献报道的术后发生EEM的12个病例，原发肺癌位置以中央型肺癌多见（分别为83.3%和75.0%），病理类型以鳞癌多见（分别为66.7%和66.7%）^[10-13, 15]^。由于鳞癌大多数表现为中央型肺癌，因此，我们认为原发肺癌的位置，尤其是中央型肺癌，是影响EEM发生的最重要因素。EEM多见于气管和左右主支气管，在支气管镜下表现为局限性支气管黏膜异常或结节/息肉样病变，这些病灶与肺癌原发病灶并无解剖连续性。这提醒支气管镜检查的操作者，对于中央型鳞癌患者在术前支气管镜检查时，不应只关注于肺癌原发病灶的活检取材，而忽视了其他微小的黏膜异常，否则有可能成为术后肺癌早期复发的隐患。

文献检索到的肺癌术后发现EEM的12例患者，大多数（75.0%）毫无症状，只是常规复查CT时发现气道病变才得以确诊，转移病灶多位于气管和主支气管^[10, 11, 13, 14]^，CT影像表现为气管、支气管管壁不规则增厚或者管腔内的突起结节^[10-17]^。Youn等^[13]^报道了1例右中叶鳞癌术后6个月时随诊CT可见气管内壁上1个很小的结节，误以为痰栓，6个月后复查，该结节明显变大，进一步行支气管镜检查证实为EEM。这也提醒我们，对于肺癌术后CT随访时应格外关注气管和左右主支气管有无异常，有助于早期发现EEM。

此外，本文有一定的局限性：①单中心研究，样本量小，存在选择偏倚可能；②肺癌原发灶和EEM是否存在双原发癌的可能性？考虑到其中大多数为非腺癌，都有很明显的肺癌原发灶，而转移灶比原发病灶要小很多。因此，我们认为双原发癌的可能性很小。

总之，EEM是肺癌的一种少见的转移方式，临床罕见，CT和支气管镜下多表现为肺癌原发病灶之外的气管和/或支气管结节或息肉样病变，可发生于初诊时，也可发生于手术切除后，预后很差。晚期中央型鳞癌发生EEM更多见，值得我们进一步关注。
